# Independent and combined effects of dietary iron composition and selected risk factors on the risk of NAFLD in a Chinese population

**DOI:** 10.1038/s41598-019-40449-1

**Published:** 2019-03-11

**Authors:** Xian-E. Peng, Shang-Hua Xu, Wenjuan Liu, Zhijian Hu, Zheng Lin, Xu Lin

**Affiliations:** 1 Department of Epidemiology and Health Statistics, Fujian Provincial Key Laboratory of Environment Factors and Cancer, School of Public Health, Fujian Medical University, Fujian, China; 2 Key Laboratory of Ministry of Education for Gastrointestinal Cancer, Fujian Medical University, Fujian, China; 3 Department of Cardiology, Affiliated Nanping First Hospital, Fujian Medical University, Nanping, China

## Abstract

Iron is an essential mineral required for most forms of life. However, very little is known in relation to the different forms of dietary iron on the development of NAFLD. The aims of this study were to investigate the effects of iron intake from different food types on risk of NAFLD and whether this effect may be modified by other factors. We conducted a hospital-based case–control study including 1,273 NAFLD cases and 1,273 gender and age-matched controls. We conducted in-person interviews while participants completed a questionnaire on food habits. We assessed animal- and plant-derived intake of iron and fat. We observed that animal-derived iron intake (>4.16 mg/day) was positively associated with augmented NAFLD risk in a Chinese population (OR_adjusted_ = 1.66 in the highest quartile compared with the lowest, 95% confidence interval [CI] = 1.01–2.73). In contrast, a high consumption of iron (>16.87 mg/day) from plant-based foods was associated with a decreased NAFLD risk (ORadjusted = 0.61 in the highest quartile compared with the lowest; 95% CI = 0.40–0.935). In addition, high intake of fat or being overweight may exacerbate this effect. Reduced consumption of iron and fat from animal sources could reduce NAFLD risk, as would weight loss.

## Introduction

Non-alcoholic fatty liver disease (NAFLD) has been defined as the hepatic accumulation of lipids (mainly triglyceride) in the absence of significant alcohol intake (<20 g/day), or other secondary causes. It encompasses a spectrum of disease ranging from uncomplicated steatosis to non-alcoholic steatohepatitis (NASH), and augments the risk of cirrhosis and hepatocellular carcinoma^1,2^. With the escalating incidence of obesity and unhealthy dietary patterns worldwide, NAFLD is currently recognized as a major health burden^3,4^. However, the pathogenesis of NAFLD remains to be elucidated. The pathophysiological mechanisms for the development and progression of NAFLD are both complex and multi-factorial. A number of parallel factors (including insulin resistance, adipose-driven hormones, nutritional factors, gut flora, and genetic factors), all act together in genetically predisposed individuals with induction of NAFLD^5^. Recently, interest has focused on the association of iron overload and NAFLD^6–8^.

Iron is an essential mineral for most forms of life. Its importance lies in its ability to mediate electron transfer and it plays a vital role in a number of essential biological functions, including transport of oxygen and cell respiration. However, iron is also potentially toxic, as it can act as a pro-oxidant. Iron has been reported as the initial precipitant in promoting liver steatosis through lipid interference^9^ or glucose metabolism^10^. Excess iron is associated with oxidative stress and leads to fat peroxidation^11,12^, B-cell dysfunction, and impaired glucose metabolism^13^. In accordance with this, previous studies have reported that approximately one third of patients with NAFLD exhibit disturbed iron homeostasis as demonstrated by high serum ferritin with normal or mildly elevated transferrin saturation^14^. While iron depletion has been shown to insulin resistance, it has also been associated with improvement in liver damage in patients with NAFLD^15,16^. Furthermore, hepatic iron may be a determinant of serum ferritin in NAFLD patients^17^. Recently, a meta-analysis revealed that serum ferritin was increased in NAFLD patients (NAFL and/or NASH) compared with control patients^18^. However, very little is known in relation to the different forms of dietary iron on the development of NAFLD in populations. Dietary iron comes in two different forms; non-heme and heme iron, both which can be derived from multiple sources. Heme iron mainly exists in animal-based products, while in both plant and animal-based foods is present as non-heme iron^19^. Heme iron is the less prevalent form of dietary iron, however, it is absorbed four to five times more easily than non-heme iron^20^, and it has been suggested that in a typical western diet the heme pool contributes up to 50% of the total iron^21^. Results from a recent epidemiological study showed that red meat consumption was associated with NAFLD risk^22^, and heme iron has partly attributed to this association^23^. Here, we explored the the effects of intake of iron from several food sources on NAFLD risk, and interactions between iron from the diet and other established risk factors of NAFLD. We did this by performing a large case-control study among a population in China, where foods have yet to be fortified with iron.

## Results

### Selected characteristics of study participants

Cases and controls differed only in: some demographic factors, established risk factors for NAFLD, and dietary factors (Table 1). There were no significant differences in gender, age, and marital status between the two groups. Compared to controls, NAFLD patients tended to be less educated, were more likely to smoke, rarely drank alcohol, and had lower income. Cases had higher blood pressure, increased body mass index (BMI), higher fasting plasma glucose (FPG), increased uric acid (UA), triglyceride (TG), total cholesterol (TC), aspartate aminotransferase (AST), alanine aminotransferase (ALT), and ApoB but had lower levels of HDL-C and ApoA (each *P* < 0.05). Finally, total fat intake was significantly greater among NAFLD patients than among controls.

**Table 1:** Selected characteristics of study participants.

Variables	Cases (n = 1273)		Controls (n = 1273)		*P* value
	%	Mean (SD)	%	Mean (SD)	
Sex (Male)	70.60		70.60		1.00
Income (RMB/Mon)					<0.05
<2000	53.18		41.48		
≥2000					
Marriage (Married)	91.28		86.57		<0.001
Education					0.007
≤6 years	65.32		59.48		
≤9 years	12.78		13.85		
>9 years	21.90		26.67		
Smoking (Yes)	29.54		22.78		<0.001
Light alcohol drinking (Yes)	26.79		21.37		0.001
Physical activity (Yes)	54.05		54.75		
Age (years)		45.81 ± 11.95		45.19 ± 12.23	0.194
AST (IU/L)		25.26 ± 14.76		23.48 ± 23.02	0.002
ALT (IU/L)		33.74 ± 29.29		25.12 ± 51.08	0.001
TC (mmol/L)		5.32 ± 0.97		5.08 ± 1.06	0.001
TG (mmol/L)		2.00 ± 1.31		1.33 ± 0.91	0.001
FPG (mmol/L)		5.89 ± 7.07		5.36 ± 1.16	0.009
HDL-c (mmol/L)		1.29 ± 0.41		1.44 ± 0.51	0.001
LDL-c (mmol/L)		3.66 ± 3.80		3.62 ± 4.46	0.797
SBP (mmHg)		126.34 ± 13.67		118.94 ± 12.89	0.001
DBP (mmHg)		81.78 ± 10.06		77.35 ± 26.60	0.001
BMI (kg/m^2)^		25.24 ± 2.81		22.34 ± 2.41	0.001
ApoA1 (mmol/L)		1.25 ± 0.48		1.29 ± 0.19	0.005
ApoB (mmol/L)		1.02 ± 0.21		0.95 ± 0.22	0.005
UA (mmol/L)		376.31 ± 87.12		333.16 ± 82.93	0.001
**Dietary**					
Total energy (kcal/day)		1631.54 ± 486.74		1596.69 ± 487.74	0.071
Total fat (mg/day)		46.65 ± 20.94		43.99 ± 21.22	0.001
Dietary calcium (mg/day)		406.79 ± 159.74		409.71 ± 156.45	0.640

Continuous variables are expressed as the mean (SD); categorical variables are expressed as percentages; n: number of individuals; SD: standard deviation.

a: obtained by the Pearson Chi-square test; *b*: obtained by independent samples *t*-tests.

### The associations of dietary iron intake with NAFLD

The association of dietary iron consumption and risk of NAFLD is presented in Table 2. Total daily iron intake and plant-derived iron intake (non-heme iron) was similar in the two groups but daily intake of animal-derived iron (heme iron) in patients with NAFLD [(7.84 ± 4.11) mg/d] was significantly higher (*P* = 0.013) than in controls [(7.24 ± 4.04) mg/d]. Considering that alcohol intake impacts upon the severity of steatosis and also on circulating iron levels, it is possible that alcohol inhibits hepcidin synthesis and thus ameliorates dietary iron absorption. Therefore, subjects who consumed alcohol to excess were excluded. A similar positive association between iron derived from animal products and NAFLD was observed among subjects without alcohol consumption (shown in Table S1). In order to adjust for potential confounding factors, controls were categorized into quartiles by consumption of total iron, heme iron, and non-heme iron (Table 3). Elevated total iron consumption was not associated significantly with NAFLD risk. However, clear differences were observed in the effects of iron derived respectively from plant and animal sources. After adjusting for potential confounders, high consumption of iron (>4.16 mg/day) from animal-sourced foods was associated strongly with increased risk of NAFLD among all participants (OR Q4 vs. Q1 = 1.66; 95% CI = 1.01–2.73; *P*_trend_ = 0.003). High consumption of iron (>16.87 mg/day) from plant-based products was associated with reduced NAFLD risk (OR Q4 vs. Q1 = 0.61; 95% CI = 0.40–0.94; *P*_trend_ = 0.013). When the fully adjusted model was stratified by gender, a significant positive association was demonstrated between animal-derived iron and NAFLD, and this effect was observed only among men (OR = 2.29; 95% CI = 1.27–4.13 in the highest quartile compared with the lowest; *P*_trend_ < 0.001). However, a significant inverse association was found between plant-derived iron and NAFLD, which was observed only for female (OR_adjusted_ = 0.33, 95% CI = 0.15–0.71 in the highest quartile compared with the lowest, *P*_trend_ = 0.043). There was no evidence of effect modification by age for the association between total dietary iron, heme iron, or non-heme iron intake and NAFLD (data not shown).

**Table 2:** The association between dietary iron intake and NAFLD.

Dietary iron (mg/d)	NAFLD patients	controls	t	*P*
Total iron	22.05 ± 7.18	21.81 ± 7.08	1.16	0.245
Heme-iron	7.84 ± 4.11	7.24 ± 4.04	**2.48**	**0.013**
Non-heme iron	14.21 ± 6.00	14.0.57 ± 5.81	0.284	0.776

Data are represented as the mean ± SD and were analyzed using the student *t*-test.

**Table 3:** Association of NAFLD risk with dietary iron intake.

Daily iron intake	Q 1	Q 2	Q 3	Q 4	P_trend_
**All (cases: 1273, controls:1273)**					
Total iron					
OR1 (95% CI)	1.00	0.80 (0.62–1.02)	1.25 (1.00–1.56)	1.07 (0.856–1.35)	0.098
OR2 (95% CI)	1.00	0.65 (0.48–0.88)	1.13 (0.85–1.51)	0.90 (0.68–1.20)	0.777
OR3 (95% CI)	1.00	0.65 (0.47–1.06)	1.13 (0.82–1.57)	0.88 (0.59–1.33)	0.687
**Animal source iron**					
OR1 (95% CI)	1.00	1.41 (1.11–1.78)	1.57 (1.25–1.98)	1.67 (1.24–2.24)	0.001
OR2 (95% CI)	1.00	1.36 (1.02–1.81)	1.78 (1.33–2.39)	1.56 (1.08–2.26)	0.001
OR3 (95% CI)	1.00	1.36 (1.01–1.83)	1.83 (1.30–2.57)	1.66 (1.01–2.73)	0.003
**Plant source iron**					
OR1 (95% CI)	1.00	1.14 (0.91–1.43)	0.95 (0.76–1.19)	0.86 (0.68–1.10)	0.116
OR2 (95% CI)	1.00	1.11 (0.83–1.50)	0.80 (0.60–1.06)	0.68 (0.51–0.60)	0.005
OR3 (95% CI)	1.00	1.04 (0.76–1.43)	0.76 (0.55–1.04)	0.61 (0.40–0.94)	0.013
**Men(cases:899, controls: 899)**					
Total iron					
OR1 (95% CI)	1.00	0.86 (0.63–1.18)	1.39 (1.05–1.84)	1.20 (0.91–1.58)	0.031
OR2 (95% CI)	1.00	0.71 (0.47–1.06)	1.35 (0.94–1.94)	1.11 (0.78–1.57)	0.118
OR3 (95% CI)	1.00	0.77 (0.50–1.17)	1.47 (0.98–2.21)	1.34 (0.81–2.21)	0.051
**Animal source iron**					
OR1 (95% CI)	1.00	1.65 (1.21–2.25)	1.98 (1.46–2.68)	2.02 (1.41–2.89)	<0.001
OR2 (95% CI)	1.00	1.57 (1.07–2.30)	2.24 (1.52–3.31)	1.84 (1.17–2.90)	0.001
OR3 (95% CI)	1.00	1.64 (1.10–2.42)	2.53 (1.64–3.90)	2.29 (1.27–4.13)	<0.001
**Plant source iron**					
OR1 (95% CI)	1.00	1.24 (0.94–1.63)	0.94 (0.71–1.25)	0.94 (0.70–1.25)	0.516
OR2 (95% CI)	1.00	1.20 (0.82–1.74)	0.78 (0.54–1.12)	0.88 (0.61–1.27)	0.277
OR3 (95% CI)	1.00	1.22 (0.82–1.81)	0.78 (0.53–1.21)	0.93 (0.54–1.59)	0.477
**Women((cases:374, controls:374))**					
Total iron					
OR1 (95% CI)	1.00	0.77 (0.51–1.15)	1.05 (0.68–1.66)	0.65 (0.42–1.02)	0.167
OR2 (95% CI)	1.00	0.67 (0.41–1.10)	0.68 (0.38–1.22)	0.56 (0.32–0.99)	0.035
OR3 (95% CI)	1.00	0.50 (0.24–1.034)	0.78 (0.44–1.34)	0.39 (0.1743–1.01)	0.022
**Animal source iron**					
OR1 (95% CI)	1.00	1.11 (0.76–1.62)	0.99 (0.66–1.48)	1.22 (0.64–2.34)	0.167
OR2 (95% CI)	1.00	1.19 (0.75–1.87)	0.98 (0.58–1.68)	1.47 (0.67–3.23)	0.526
OR3 (95% CI)	1.00	1.00 (0.59–1.60)	0.75 (0.38–1.49)	0.99 (0.33–2.94)	0.619
**Plant source iron**					
OR1 (95% CI)	1.00	0.88 (0.54–1.45)	0.94 (0.63–1.40)	0.54 (0.35–0.85)	0.049
OR2 (95% CI)	1.00	0.62 (0.32–1.21)	0.81 (0.50–1.32)	0.36 (0.21–0.63)	0.002
OR3 (95% CI)	1.00	0.50 (0.24–1.03)	0.78 (0.45–1.35)	0.33 (0.15–0.71)	0.043

Odds ratios (ORs) and their 95% confidence intervals (95% CIs) are presented.

OR_1_: adjusted for age, gender, education, family income, regular exercise, smoking and moderate alcohol consumption.

OR_2_: adjusted for BMI, systolic blood pressure, TC, LDL-C, HDL-C, TGs, FPG, UA, ApoA, ApoB, as well as variables for OR_1_.

OR_3_: adjusted for dietary total fat, total energy intake and dietary calcium intake in addition to variables for OR_2_.

### Clinical parameters and their association with different quartiles of animal-derived iron from foods in NAFLD patients and controls

We assessed the relationship between quartiles of animal-derived iron, BMI, fasting plasma glucose (FPG), and serum lipids in NAFLD and control subjects, respectively. Among NAFLD patients, subjects with higher animal-derived iron intake tended to be associated with higher levels of BMI, UA, and ALT. Whereas in controls, subjects with higher animal-derived iron consumption tended to be associated with higher levels of serum UA and lower levels of HDL-C (shown in Table S2).

### The combined effects of animal-derived iron and selected risk factors on NAFLD risk

We also explored the combined effects of animal-derived iron and established risk factors of NAFLD (Table 4). We did not observe significant interactions between animal-derived iron, moderate alcohol consumption, or smoking on NAFLD risk were observed. However, animal-derived iron appeared to have a synergistic effect with dietary fat intake (*P* for interaction = 0.041) and being overweight or obese (*P* for interaction = 0.006) in precipitating NAFLD risk. Specifically, subjects with the highest intake of animal-derived iron and higher levels of total dietary lipid, corresponding to >9.71 mg iron/day and >44 mg lipid/day from the diet had the greatest risk of NAFLD compared with subjects ingesting low levels of animal-derived iron and total fat (OR _adjusted_ = 2.08, 95% CI:1.50–2.89). Similarly, subjects with the highest intake of animal-derived iron and BMI ≥24 kg/m^2^ had the highest NAFLD risk compared with subjects consuming low levels of iron derived from animals and BMI < 24 kg/m^2^ (adjusted OR = 9.15, 95% CI: 5.96–14.07).

**Table 4:** Interaction effects of animal iron and selected risk factors on the risk of NAFLD.

Exposures		Quartiles of animal iron intake				*P-*value for multiplicative interactions
		Q1	Q2	Q3	Q4	
**Smoking** ^**b**^						
No	Cases/controls a	193/250	211/232	196/240	263/242	0.119
	OR (95%CI)	1.00	1.31 (0.97–1.78)	1.46 (1.04–2.05)	1.51 (1.06–2.15)	
Yes	Cases/controls a	47/68	103/87	84/77	176/77	
	OR (95%CI)	0.85 (0.52–1.40)	1.45 (0.96–2.19)	1.73 (1.11–2.72)	3.35 (2.20–5.12)	
**Moderate alcohol drinker** ^**c**^						
No	Cases/controls a	185/254	218/221	200/267	263/179	0.628
	OR (95%CI)	1.00	1.43 (1.05–1.94)	1.38 (0.99–1.933)	2.14 (1.49–3.08)	
Yes	Cases/controls a	55/64	96/98	80/50	176/140	
	OR (95%CI)	1.04 (0.65–1.67)	1.36 (0.91–2.02)	2.43 (1.52–3.89)	1.76 (1.19–2.59)	
**Dietary fat intake** ^**d**^						
Low	Cases/controls a	234/315	251/265	70/68	17/14	0.041
	OR (95%CI)	1.00	1.35 (1.02–1.78)	1.82 (1.18–2.80)	1.78 (0.75–4.22)	
High	Cases/controls a	6/3	63/54	210/249	422/305	
	OR (95%CI)	4.48 (0.74–27.14)	1.82 (1.14–2.91	1.56 (1.12–2.17)	2.08 (1.50–2.89)	
**Overweight or obesity** ^**e**^						
No	Cases/controls a	135/275	167/283	173/298	132/259	0.006
	OR (95%CI)	1.00	1.33 (0.96–1.82)	1.74 (1.23–2.47)	1.31 (0.89–1.94)	
Yes	Cases/controls	105/42	147/36	104/19	307/60	
	OR (95%CI)	4.31 (2.75–6.76)	7.53 (4.74–11.96)	10.35 (5.77–18.56)	9.15 (5.96–14.07)	

^a^The number of cases/controls in each cell is followed by the odds ratio (OR) and 95% confidence interval (95% CI) for NAFLD.

^b^ORs and P-values for multiplicative interactions are adjusted for age, gender, family income, education, moderate alcohol consumption, BMI, regular exercise, total energy intake, total fat intake, systolic blood pressure, and biochemistry.

^c^ORs and P-values for multiplicative interactions are adjusted for age, gender, family income, education, smoking, BMI, regular exercise, total energy intake, total fat intake, systolic blood pressures, and biochemistry.,

^d^ORs and P-values for multiplicative interactions are adjusted for age, gender, family income, education, smoking, moderate alcohol consumption, BMI, regular exercise, total energy intake, systolic blood pressure, and biochemistry.

^e^ORs and P-values for multiplicative interactions are adjusted for age, gender, family income, education, smoking, moderate alcohol consumption, regular exercise, total energy intake, total fat intake, systolic blood pressure, and biochemistry.

## Discussion

In this large case-control study of a Han Chinese population, high daily consumption of iron (>4.16 mg/day) derived from animal sources was associated with significantly augmented risk of NAFLD, particularly in men. However, a significant inverse association was found between a high daily intake of plant-source iron (>16.87 mg/day) and NAFLD, particularly among women. We also observed a statistically significant synergistic interaction between animal-derived iron and BMI ≥24 kg/m^2^. To our knowledge, this is the first case-control study to ever evaluate the independent and combined effects of different iron forms from dietary and selected risk factors on the risk of NAFLD.

Iron is an essential mineral, which is required for vital functions throughout the body. It is utilized as an enzyme cofactor, involved in oxygen transport, plays an essential role in ATP generation, and it supports growth^24^. An excess of iron is also, however, a potent cause of cellular injury from oxidative stress due to the generation of ROS mediated by the Fenton reaction^25^. A strong association between excess iron that is not related to several metabolic syndrome (MetS) features and hereditary hemochromatosis (HHC), has been demonstrated^26–29^. However, most studies have focused on serum iron markers (ferritin or hepcidin) and NAFLD risk, where disturbed serum levels of these elements were induced by conditions such as insulin resistance and hyperglycemia^30,31^. Thus, serum levels of these elements can be both contributors to the development of NAFLD and/or the result of metabolic distress in NAFLD. However, in the present work, we evaluated the association between intake of iron from different food sources with risk of NAFLD, rather than serum levels of iron. After adjusting for potential confounders, we found that animal-derived iron was associated strongly with increased risk of NAFLD among all participants, while high consumption of iron from plant-based products was associated with decreased NAFLD risk. Our observations for dietary iron largely reflect those found in another Chinese population conducted by Zheng *et al*.^32^, who demonstrated a significantly positive association between dietary heme-iron intake and NAFLD. However, dietary intake of non-heme iron was not associated with NAFLD. Our findings were also supported by experimental results noted in mice where a high-iron diet was positively associated with elevated hepatic cholesterol synthesis leading to hyperglycemia, hyperinsulinemia, and IR, which are risk factors for NAFLD^33,34^. In addition, NAFLD has been regarded as a “hepatic manifestation of the metabolic syndrome (MetS)”. Therefore, our findings were indirectly supported by several studies that revealed a positive association between heme iron consumption and risk of type 2 diabetes mellitus and cardiovascular disease; these share common metabolic parameters with NAFLD^29,35–37^. The unusual effects of animal-derived iron and plant-iron consumption on NAFLD risk may be explained by the differences in bioavailability and their effects on iron stores within the body^38^. Iron obtained from animal products mainly consists of heme iron, which has high bioavailability, and is the main contributor to stored body iron in humans. Non-heme iron is less bioavailable but also mainly obtained from fruit, vegetable, whole grains, and fortified cereals. These are commonly considered to be beneficial foods that contain some healthy components such as plant proteins, fiber and others antioxidants. Furthermore, dietary patterns may alter the gut microbiota^39^, which contributes to pathogenesis of NAFLD through multiple mechanisms^40^.

Previous studies on iron-NAFLD risk either assessed one gender or did not provide a stratified analysis^32^. In the present study, when the model was stratified by gender, a significant positive association was found between animal-derived iron and NAFLD only for men. In contrast, a significant inverse association was found between plant-derived iron and NAFLD only for women. The gender difference observed in this study may be explained by the heterogeneity in iron absorption. Studies have reported that women generally have higher iron requirements, and they absorb approximately twice as much iron as men from the same dietary intake of this mineral^41^. Nonetheless, further studies are needed to validate the gender heterogeneity in the aforementioned association between heme iron and NAFLD, and potential mechanisms should be explored further.

Increased hepatic iron associated with the MetS has been commonly observed as a syndrome and has been named as ‘dysmetabolic iron overload syndrome^42,43^. In this study, we also evaluated the influence of animal-derived iron on metabolic syndrome components and we observed that subjects with higher animal-derived iron intake tended to be associated with higher levels of BMI and UA among NAFLD patients. In controls, subjects with higher animal-derived iron intake tend to be associated with higher levels of serum UA and lower levels of HDL-C. Previous studies have reported that high levels of BMI and serum UA were associated with NAFLD risk. Therefore, we speculate that high animal-derived iron intake is associated with NAFLD risk by increased BMI and/or serum UA. Nevertheless, more studies are required to confirm our findings.

The underlying mechanisms for the development of NAFLD are complex and multi-factorial. We also found that animal-derived iron appeared to have a combined effect with dietary fat intake and being overweight or obese with respect to increased NAFLD risk. We postulate that one possible mechanism for the interaction between iron and fat is that particular fats and fatty acids might significantly enhance transport of reactive forms of iron into cells^44^. Another reason for the interaction may be explained by results from an *in vivo* study, which showed that a high-fat diet and additional iron acted synergistically in exacerbating hyperglycemia, hyperinsulinemia, and IR. Furthermore, such a diet/supplementation may induce synthesis of lipids, and thereby increase hepatic TG and cholesterol levels, resulting in hepatic steatosis^33,34^. In addition, we also found that subjects with BMI ≥24 kg/m^2^ experienced an enhanced risk of NAFLD associated with animal-derived iron intake than their non-overweight peers. The mechanisms underlying the effect modification by BMI in this study have yet to be elucidated; therefore, future studies are needed to evaluate the potential mechanisms.

Our study had both strengths and limitations. The strength of our study was the large sample size, which could have reduced type II errors. Secondly, detailed collection of data through face-to-face interviews using a FFQ and extensive information on anthropometrics and lifestyle factors were also collected in this study, which allowed us to potentially adjust for confounding factors. The study had enough power to allow investigation of interactions between animal-derived iron and total lipid; there is a plausible mechanism for such interactions. Nonetheless, several limitations of our study require consideration when interpreting study findings. First, selective participation and recall bias are potential concerns in our study as with other epidemiologic studies of this nature. Second, because dietary iron intake was self-reported, we cannot exclude the possibility of reporting bias. Therefore, our study can only provide etiological clues in exploring the association between dietary heme iron intake and NAFLD. It cannot confirm whether the difference in heme iron intake between NAFLD subjects and controls is really biologically relevant. Third, we found it difficult to obtain samples from individuals who attended for a regular medical health check-up. Thus, we only examined dietary intake rather than measurement of circulating iron levels, and so we cannot accurately predict what level of iron was absorbed. Fourth, as with all observational studies, although we adjusted for known confounders, it is not possible to exclude others confounding of unknown covariates. Finally, our study was performed on a health examination of the Han Chinese population. Therefore, extrapolation of our findings to the general population or other ethnicities should be done with caution. Further population-based studies are warranted to verify our results, especially those that include different ethnic groups or populations that eat predominantly plant-based products e.g. vegetarians.

## Conclusions

In this Chinese population, we found that high consumption of animal-derived iron was associated with significantly increased risk of NAFLD, particularly among men. This effect may be augmented by high total fat intake or being overweight. While confirmation of these findings in other populations is required, and further exploration of possible underlying biological mechanisms of observed associations is warranted, our study suggests that animal-derived iron and fats or BMI have combined effects in the development of NAFLD. We suggest reductions in consumption of animal-derived iron and fat (or weight loss) to reduce NAFLD risk.

## Methods

### Study design

We performed a case-control study for NAFLD in health examination centers at two teaching hospitals of Fujian Medical University (the Union Hospital and Affiliated Nanping First Hospital, China), from June of 2011 to May of 2017. As described in our previous work^45^, criteria for inclusion of cases in the study was permanent residency in urban Fujian being 20–74 years of age, no secondary causes of steatosis, including alcohol misuse in the past year (weekly ethanol consumption ≥140 g for men, or ≥70 g for women), total parenteral nutrition, infection with hepatitis B or hepatitis C virus, or the use of drugs known to precipitate hepatic steatosis prior to diagnosis (cases) or study enrollment (controls). Incident cases were diagnosed by liver ultrasonography using established criteria^46^. Controls with no liver steatosis by ultrasonography were randomly selected from the same center during the same study period. Eligibility criteria for controls were identical to those for cases with the exception of liver steatosis diagnosis. Controls were frequency matched to cases on age (within 5-yr intervals), gender, ethnicity, occupation and region of origin. Additionally, cases and controls with any of the following were excluded from the study: type II diabetes, hypertension, hyperlipidemia, autoimmune hepatitis, drug-induced liver disease, primary biliary cirrhosis, and primary sclerosing cholangitis. 1,273 eligible cases and controls were recruited to the study and completed an interview. All enrolled subjects were of Chinese Han ethnicity. No significant differences were found for the mean age and gender distribution between cases and controls. All subjects who participated in this study provided written informed consent and the study was approved by the local ethics committees of Fujian Medical University. In addition, all methods were performed in accordance with the relevant guidelines and regulations of the University.

### Physical, anthropometric, and biochemical evaluation

After an overnight fast, all subjects underwent a complete physical examination in the morning. The examination included anthropometric measurements, abdominal ultrasonography, and biochemical determinations. Body weight, height, diastolic arterial blood pressure (DABP) and systolic arterial blood pressure (SABP) were measured using standardized procedures^47^. Body mass index (BMI) was calculated as weight in kilograms divided by height in meters squared. As described in our previous work^48^, hypertension was defined as systolic blood pressure ≥140 mmHg and/or diastolic blood pressure ≥90 mmHg, or if the subject was taking anti-hypertensive medications. Venous blood samples were taken immediately after the anthropometric examinations. Total cholesterol (TC), low-density lipoprotein cholesterol (LDL-C) and high-density lipoprotein cholesterol (HDL-C), TGs, apolipoprotein A (ApoA1), apolipoprotein B (ApoB), serum uric acid (UA), fasting plasma glucose (FPG), alanine aminotransferase (ALT), and aspartate aminotransferase (AST) were measured by standard clinical laboratory techniques.

### Dietary Assessment

Dietary information on typical food consumption was collected by an evaluated 125- item semiquantitative food frequency questionnaire (SFFQ), which had been specifically developed and validated for the southern Chinese population^49^. Participants were asked to estimate information on the average frequency of consumption of selected foods (rarely, < once/month, 1–3 times/month, 1–2 times/week, 3–4 times/week, 5–6 times/week, once/day, twice/day and >twice/day). Ignoring any recent changes, serving sizes in lians (1 lian equals 50 g) or jins (where 1 jin equals 500 g) over the previous year were considered. These frequencies were then multiplied by 0, 0.03, 0.07, 0.22, 0.50, 0.79, 1, 2, 3/day, respectively to obtain the intake of each food item. When seasonal foods were assessed, subjects described their intake based on availability and number of months per year in which they consumed the food. A lifestyle questionnaire was used to collect information relating to socio-demographic characteristics and lifestyles. The information collected included age, gender, family income, marital status, education level, tea drinking, smoking status, light alcohol consumption, physical activity, and medical history.

### Assessment of iron intake

The quantity of iron for each food item was calculated from Chinese food composition tables^50^. For each food and beverage queried, the product of daily frequency scores and standard serving size (in grams) was multiplied by the iron content per 100 g of each food to obtain the daily iron intake. Daily iron intake from various foods was obtained by adding up all the food items. We did not have sufficient data on iron supplementation therefore we could not measure supplemental iron intake. We similarly estimated daily total energy intake (kcal) by calculating the energy intake for individual food items. We also used the FFQ to assess heme iron intake, which was estimated by calculating iron intake that was exclusively derived from animal foods. These included beef, pork, sausage, chicken, liver, fish, dried fish, and boiled fish paste. Non-heme iron intake was calculated as total iron minus heme iron intake.

### Statistical analysis

χ^2^ tests were used to compare study participant characteristics, including gender, family income, marital status, education level, tea drinking, smoking status, alcohol consumption, and physical activity according to NAFLD status. Independent sample t-tests were used to compare mean values according to NAFLD status. Consumption of total iron, heme iron, and non-heme iron was categorized among controls in quartiles. The association between NAFLD risk and each quartile of iron intake was determined by unconditional logistic regression models [calculating odds ratios (OR) and their 95% confidence intervals (CIs)], using the lowest quartile as the reference category. Independent risk factors for NAFLD—age, gender, marital status, family income, educational attainment, body mass index (BMI), regular physical activity (yes/no), current cigarette smoking, biochemistry, total dietary fat intake, and total energy intake—were included in all models. By conducting stratified analyses and evaluating interaction terms, we evaluated whether associations between dietary animal-derived iron consumption and NAFLD risk were modified by other established risk factors of NAFLD. These factors included BMI (<24 or ≥24 kg/m^2^), moderate alcohol consumption (yes or no), current cigarette smoking (yes or no), and dietary fat intake (low or high). All data analyses were performed using IBM SPSS Statistics Release Version 19.0.0.1 (IBM SPSS, 2010, Chicago, IL).

## Supplementary information

supplementary tables
