# Supplementary material for: Independent and combined effects of dietary iron composition and selected risk factors on the risk of NAFLD in a Chinese population
Source: Sci Rep. 2019 Mar 11;9:4069. doi: 10.1038/s41598-019-40449-1 (PMC6411980; doi:10.1038/s41598-019-40449-1)
Supplement: Supplementary file 1 — supplementary tables [file 41598_2019_40449_MOESM1_ESM.pdf]

Independent and combined effects of dietary iron composition and selected risk factors on the risk of NAFLD in a Chinese population

Xian-E Peng[1, 2*],Shang-Hua Xu[3] ,Wenjun Liu[1], Zhijian Hu[1], Zheng Lin[1], Xu Lin[2]

[1]Department of Epidemiology and Health Statistics, Fujian Provincial Key Laboratory of Environment Factors and Cancer, School of Public Health, Fujian Medical University, Fujian, China.

[2]Key Laboratory of Ministry of Education for Gastrointestinal Cancer, Fujian Medical University, Fujian, China.

[3] Department of Cardiology, Affiliated Nanping First Hospital, Fujian Medical University, Nanping, China

§ Corresponding author. Xian-E Peng, Department of Epidemiology and Health Statistics, Fujian Provincial Key Laboratory of Environment Factors and Cancer, School of Public Health, Fujian Medical University, Fuzhou,China, 350108

Phone: +86-591 22862648

E-mail: fmuxe@163.com

Dietary iron (mg/d)	NAFLD patients	controls	t	*P*
Total iron	21.46±7.13	21.24±7.08	0.68	0.499
Heme-iron	7.55± 3.80	6.99± 4.00	**3.00**	0.003
Non-heme iron	13.91±5.86	14.25±5.55	-1.26	0.208

Table S1. The association between dietary iron intake and NAFLD among subjects without alcohol consumption

Quantitative phenotype	NAFLD					Control				P^a
	Q1 (n=240)	Q2 (n=314)	Q3 (n=528)	Q4 (n=191)	P^a	Q1 (n=318)	Q2 (n=319)	Q3 (n=473)	Q4 (n=163)	
BMI (kg/m^2)	24.67±2.77	24.95±2.86	25.58±2.85	25.49±2.52	<0.01	22.31±2.57	22.39±2.32	22.31±2.42	22.42±2.20	0.93
FBS (mmol/L)	5.83±1.45	5.67±1.43	6.19±10.83	5.50±0.59	0.61	5.48±1.48	5.27±1.15	5.39±0.84	5.24±1.23	0.07
TC(mmol/L)	5.26±0.85	5.28±0.99	5.40±1.05	5.22±0.83	0.09	5.18±0.95	5.07±1.20	5.12±1.07	4.76±0.93	<0.01
TG(mmol/L)	2.05±1.26	2.08±1.36	1.94±1.33	1.95±1.21	0.40	1.40±1.02	1.44±1.12	1.20±0.59	1.35±0.93	<0.01
HDL-C (mmol/L)	1.31±0.22	1.30±0.26	1.28±0.56	1.29±0.28	0.34	3.75±4.56	3.90±5.74	3.57±3.53	3.00±3.73	<0.01
LDL-C (mmol/L)	3.60±4.12	3.82±5.01	3.74±2.82	3.28±3.38	0.45	1.35±0.23	1.35±0.32	1.54±0.71	1.54±0.41	0.19
SBP (mm Hg)	125.78±14.85	126.21±13.35	126.74±13.32	126.68±13.30	0.71	118.40±14.24	118.23±13.68	119.56±11.56	119.62±11.72	0.37
DBP (mm Hg)	81.59±9.76	82.75±9.79	81.43±9.91	81.42±10.96	0.27	76.89±10.07	76.68±9.92	78.05±9.79	77.53±10.56	0.89
AST (IU/L)	24.10±8.46	25.24±8.16	25.32±19.93	26.58±12.14	0.39	22.57±7.39	23.29±15.68	23.33±12.12	26.04±55.98	0.46
ALT (IU/L)	27.83±15.88	33.11±17.32	35.09±39.01	38.50±25.12	0.001	21.50±12.42	23.39±26.03	26.48±33.48	31.6079	0.18
UA (mmol/L)	352.70±87.78	380.74±93.86	377.59±84.10	395.07±76.89	<0.01	316.56±81.96	333.36±87.14	334.69±75.77	360.68±88.95	<0.01
apoA1 (mmol/L)	1.26±0.13	1.31±0.88	1.21±0.29	1.27±0.17	0.059	1.30±0.14	1.29±0.16	1.29±0.21	1.31±0.22	0.32
apoB (mmol/L)	1.05±0.21	1.04±0.22	0.99±0.22	1.01±0.18	<0.01	1.01±0.22	0.99±0.21	0.91±0.20	0.92±0.21	<0.01

Table S2. Comparison of various quantitative phenotypes among the different quartiles of animal-derived iron in patients with NAFLD and control subjects.

Data are represented as the mean ± SD. *a*: Data were analyzed using the Kruskal–Wallis test in each group of NAFLD and control subjects.
